# Long‐term outcomes of frontline intensification in primary CNS lymphoma: A real‐world single‐center experience

**DOI:** 10.1002/cam4.5607

**Published:** 2023-01-17

**Authors:** Hao‐Yuan Wang, Ching‐Fen Yang, Chia‐Hsin Lin, Liang‐Tsai Hsiao, Po‐Shen Ko, Yao‐Chung Liu, Tzeon‐Jye Chiou, Po‐Min Chen, Jyh‐Pyng Gau, Jin‐Hwang Liu, Chia‐Jen Liu

**Affiliations:** ^1^ Division of Hematology and Oncology, Department of Medicine Taipei Veterans General Hospital Taipei Taiwan; ^2^ Faculty of Medicine, School of Medicine National Yang Ming Chiao Tung University Taipei Taiwan; ^3^ Department of Pathology and Laboratory Medicine Taipei Veterans General Hospital Taipei Taiwan; ^4^ Department of Radiation Oncology Linkou Chang Gung Memorial Hospital Medical Center Taoyuan City Taiwan; ^5^ Institute of Biopharmaceutical Sciences National Yang Ming Chiao Tung University Taipei Taiwan; ^6^ Chong Hin Loon Memorial Cancer and Biotherapy Research Center National Yang Ming Chiao Tung University Taipei Taiwan; ^7^ Institute of Public Health National Yang Ming Chiao Tung University Taipei Taiwan

**Keywords:** frontline consolidation, frontline intensification, non‐Hodgkin's lymphoma, primary CNS lymphoma

## Abstract

**Background:**

Frontline intensification (including consolidative whole‐brain radiotherapy or high‐dose chemotherapy with autologous stem‐cell transplantation after induction therapy) has been proposed to treat primary central nervous system lymphoma (PCNSL). However, no prospective randomized trials have answered whether frontline intensification can offer a survival benefit to PCNSL patients. We aim to clarify the outcomes and survival influence of frontline intensification on real‐world patients with different risk‐stratified PCNSLs.

**Methods:**

Between January 2003 and December 2016, 110 PCNSL adults were retrospectively included, and 76 patients achieved at least PR after induction therapy, including 38 patients who received frontline intensification. The median follow‐up with the 31 survivors was 7.52 years.

**Results:**

Of the 38 induction‐completed patients who had not received frontline intensification, 95% achieved post–induction therapy CR/CRu; however, all inevitably recurred. In the 38 who received frontline intensification, CR/CRu improved from 45% (pre‐frontline intensification) to 84% (post‐frontline intensification), and they achieved significantly better PFS (non‐reach vs. 522 days, *p* < 0.001) and OS (non‐reach vs. 899 days, *p* < 0.001). Additionally, patients had similar PFS and OS rates when receiving HDC‐ASCT and/or WBRT as frontline intensification. Frontline intensification significantly improved PFS and OS survival in higher‐risk patients (intermediate/high IELSG risk, MSKCC group 2/3, or Nottingham/Barcelona score ≥ 2 points) but did not improve OS in lower‐risk patients. Among the 38 patients who received frontline intensification, two had treatment‐related mortality; 14 recurred after frontline intensification. MTX‐based chemotherapy was the main salvage modality, and the median OS was 295 days after recurrence. Progressive disease and infection (especially pneumonia) are two major causes of mortality in patients who receive frontline intensification.

**Conclusions:**

When achieving CR/CRu/PR after induction chemotherapy, frontline intensification should be adopted to improve PFS and OS in real‐world PCNSL patients, especially higher‐risk patients.

## Graphical abstract

Frontline intensification significantly improves PFS and OS in induction‐completed PCNSL patients, especially those who are at higher risk. Progressive disease and infection (especially pneumonia) are two major causes of mortality in patients who receive frontline intensification.

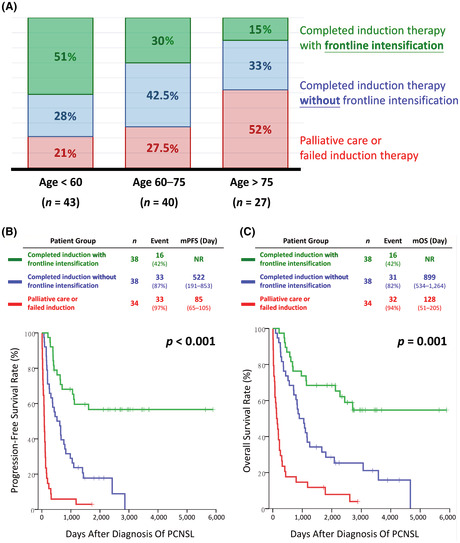

## 1 INTRODUCTION

Primary CNS lymphoma (PCNSL) represents a rare subtype of non‐Hodgkin's lymphoma (NHL), with short‐lasting remission and a poor prognosis.^1^ Several single‐arm trials have revealed an impressive therapeutic response in patients who received consolidative whole‐brain radiotherapy (WBRT) or high‐dose chemotherapy with autologous stem cell transplantation (HDC‐ASCT).^2^, ^3^, ^4^, ^5^ Although WBRT was once considered the most efficacious and universally available consolidation, there is a rising prevalence of adopting consolidative HDC‐ASCT given the neurotoxicity of WBRT. Recently, the comparable survival outcomes between patients who received consolidative WBRT and HDC‐ASCT have further been proven by two important clinical trials, IELSG‐32^6^, ^7^ and PRECIS.^8^

However, there have been few randomized controlled trials directly elucidating the survival benefits provided by consolidative WBRT or HDC/ASCT. Only one trial (G‐PCNSL‐SG‐1) compared WBRT versus watching and waiting after frontline chemotherapy, and it revealed a non‐significant trend for better PFS in the WBRT arm of the per‐protocol population, but the high rate of protocol violations (>40%) precluded making a solid conclusion.^9^ Unlike other stage IE lymphoma, PCNSL is unique regarding receiving consolidative WBRT or HDC/ASCT during frontline therapy, although PCNSL patients who have received frontline HDC‐ASCT have shown a higher mortality rate than other‐site systemic lymphoma patients.^10^

Three renowned PCNSL prognostic scoring systems using various factors have been proposed, including the International Extranodal Lymphoma Study Group (IELSG) risk score,^11^ the Nottingham/Barcelona (NB) prediction scoring system,^12^ and the Memorial Sloan Kettering Cancer Center (MSKCC) prognostic grouping.^13^ Currently, no treatment algorithm based on the above risk stratifications exists, and we are eager to validate whether frontline intensification could improve the outcome in different PCNSL risk groups.

On the other hand, real‐world patients treated outside of trials usually have a much worse outcome than patients enrolled in clinical trials because patients outside of trials have more unfavorable prognostic factors,^14^ and we are interested in whether the therapeutic outcome within PCNSL trials can be replicated in real‐world practice. Consolidation traditionally denotes treatment provided after cancer remission post‐induction therapy and is adopted as a treatment option for patients who have achieved complete response,^15^ but WBRT and HDC‐ASCT could be options for patients who have achieved partial response (PR) in real‐world clinical practice; we consequently define the WBRT and/or HDC‐ASCT that were provided to patients who achieved at least PR after induction chemotherapy as “frontline intensification.”

In this study, we aim to bridge the gap in the current evidence when treating PCNSL, clarify whether frontline intensification can provide clinical benefits in real‐world PCNSL patients, and elucidate the influence of different risk‐stratification models on frontline intensification. Accordingly, we consecutively included 110 PCNSL cases, of which 76 patients completed induction therapy and achieved at least PR. Among the 76 induction‐completed patients, 38 received frontline intensification and were regarded as the target cohort, in order to analyze their clinical characteristics and outcomes associated with frontline intensification and to determine whether frontline intensification improves the survival of higher‐risk PCNSL patients.

## 2 METHODS

### 2.1 Study population

Adults with newly diagnosed PCNSL, between January 1, 2003, and December 31, 2016, at Taipei Veterans General Hospital, were retrospectively and consecutively included. The inclusion criteria were as follows: patients had histologically proven diagnosis of non‐Hodgkin's lymphoma, lesions exclusively involved the CNS, leptomeninges, cranial nerves, and eyes, and patients had completed frontline treatment with at least partial remission. Patients with HIV or other concurrent malignancies were excluded. This study was approved by the Institutional Review Board of Taipei Veterans General Hospital (no. 2016–05‐003 BC and 2020–06‐017 AC), and the need for informed consent was waived.

### 2.2 Definition of frontline intensification

The following criteria were used to define frontline intensification: HDC‐ASCT and/or WBRT must be included in the frontline therapy; lesions must have achieved at least partial remission before starting WBRT or HDC‐ASCT; patients must have received at least four other courses of chemotherapy (not including HDC).

In patients who received frontline intensification, three subgroups were included: first, patients who received HDC‐ASCT as frontline intensification; second, patients who received WBRT as frontline intensification; third, patients who received both WBRT and HDC‐ASCT as frontline intensification.

### 2.3 Data collection

Included cases were initially categorized according to whether patients successfully completed induction therapy or not, followed by whether patients received frontline intensification or not. Among induction‐completed patients who did not receive frontline intensification, two subgroups were included: first, patients who received merely MTX‐based chemotherapy without frontline intensification; second, patients who received mainly WBRT, including eight patients who initially received ≤ three courses of chemotherapy but discontinued chemotherapy due to adverse effects or intolerance, rather than disease progression.

Risk stratification of PCNSL was defined according to the IELSG risk score,^11^ the Nottingham/Barcelona prediction scoring system,^12^ and MSKCC prognostic grouping.^13^

The response criteria of PCNSL were according to the guidelines proposed by the International Primary CNS Lymphoma Collaborative Group.^16^ Deep brain involvement is defined as the involvement of periventricular regions, basal ganglia, brainstem, and/or cerebellum.^11^

### 2.4 Statistical analysis

To compare patients with and without frontline intensification, *t*‐tests or Mann–Whitney *U* tests were used for quantitative data, Fisher exact tests for categorical data, and the Kaplan–Meier estimate and log‐rank test for survival data. The diagnosis date was defined as the date of the biopsy. Follow‐up was continued until death or dropout. The study simultaneously referred to data from the Taiwan Cancer Registry Center, of the Health Promotion Administration, as the organization recorded cancer patients' exact date of death and reported to the hospitals where patients' PCNSL were originally diagnosed at the end of the next year. Endpoints for overall survival (OS) and progression‐free survival (PFS) were defined as death due to any cause, and disease progression or death due to PCNSL, respectively.^16^

Cox regression models were used to calculate hazard ratios (HRs), and all candidate factors with a *p‐*value of ≦0.2 in the univariate analysis were subsequently entered into a multivariate regression model. The HRs of all factors are reported with the corresponding *p‐*values and 95% confidence intervals (CIs). All statistical analyses were performed using SPSS, version 22.0 (SPSS Inc.); a two‐tailed *p‐*value of <0.05 was considered statistically significant.

## 3 RESULTS

### 3.1 Clinical features of 110 intention‐to‐treat PCNSL patients

**FIGURE 1:**
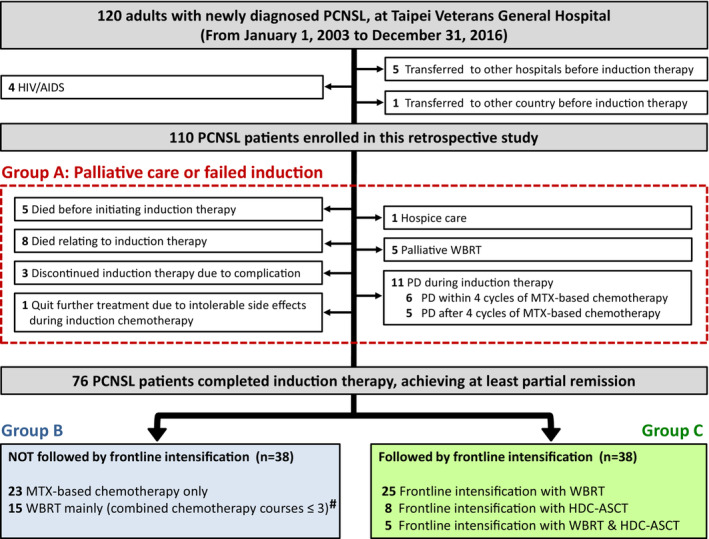
Enrollment algorithm for newly diagnosed PCNSL adults. Patients were subcategorized into three major groups: Group A, palliative care or induction failure; Group B, induction‐completed patients without frontline intensification; Group C, induction‐completed patients with frontline intensification. # Of 15 patients who received mainly WBRT, eight received MTX‐based chemotherapy before WBRT but discontinued chemotherapy due to intolerance (*n* = 2) or adverse effect (hepatitis, *n* = 3; acute renal failure, *n* = 2; bradycardia, *n* = 1), including four patients who received one cycle of chemotherapy, three patients who received two cycles, and one patient who received three cycles; the median value of cumulative dosage of MTX in these eight patients was as low as 3 g/m^2^. AIDS, acquired immune deficiency syndrome; C/T, chemotherapy; F/U, follow‐up; HDC + ASCT, high‐dose chemotherapy with autologous stem‐cell transplant; HIV, human immunodeficiency virus; MTX, methotrexate; PCNSL, primary central nervous system lymphoma; WBRT, whole‐brain radiotherapy.

As shown in Figure 1, we included 110 PCNSL cases, 31% of whom (*n* = 34) chose palliative care or had induction failure, and only 69% of whom (*n* = 76) completed induction therapy and achieved at least partial remission. Thirty‐eight patients received frontline intensification, WBRT was the most common modality of frontline intensification, and 13 patients received conditioning high‐dose chemotherapy, including BEAM (carmustine [BCNU], etoposide, cytarabine, melphalan), thiotepa plus BCNU, and BEAC (BCNU, etoposide, cytarabine, cyclophosphamide) for eight, three, and two patients, respectively.

All 110 PCNSL patients had diffuse large B‐cell lymphoma (DLBCL), their median age was 65, and the median PFS and OS were 1.1 years (400 days) and 2.3 years (833 days), respectively (Figure S1 and Table S1), after a median follow‐up of 2.3 years (833 days). Of note, the median follow‐up with the 31 survivors was as long as 7.52 years (2745 days).

### 3.2 Response comparison between induction‐completed patients with or without frontline intensification

In induction‐completed patients without frontline intensification (Table 1), although they were older, 95% achieved complete remission (CR) or unconfirmed complete remission (CRu). In contrast, only 45% of the patients who received subsequent frontline intensification achieved CR/CRu before frontline intensification; however, the CR/CRu rate was impressively elevated to 84% after frontline intensification.

**TABLE 1:** Characteristics of 110 intention‐to‐treat PCNSL patients

Characteristics	Palliative care or failed induction (*n* = 34)	Completed induction therapy^a^		*p*‐value^b^
		Without intensification (*n* = 38)	With intensification (*n* = 38)	
Age, years	72 [56–80]	66 [58–74]	57 [49–68]	0.020
≥70	18 (53)	14 (37)	8 (21)	0.206
<70	16 (47)	24 (63)	30 (79)	
Sex, male	20 (59)	21 (55)	19 (50)	0.819
ECOG				
0	3 (9)	4 (11)	10 (26)	0.168^c^
1	9 (26)	12 (32)	13 (34)	
2	10 (29)	9 (23)	7 (18)	
3	5 (15)	10 (26)	7 (19)	
4	7 (21)	3 (8)	1 (3)	
Histology: DLBCL	34 (100)	38 (100)	38 (100)	1.000
PTLD	0 (0)	0 (0)	1 (3)^d^	
Elevated serum LDH	21 (62)	14 (37)	17 (45)	0.641
Multiple brain lesions	14 (41)	21 (55)	20 (53)	1.000
Deep‐brain involvement	25 (74)	24 (63)	25 (66)	1.000
Ocular involvement	3 (9)	6 (16)	5 (13)	1.000
Leptomeningeal involvement	1 (3)	2 (5)	2 (5)	1.000
IELSG risk				
Low	0 (0)	5 (13)	5 (13)	
Intermediate	22 (65)	25 (66)	23 (61)	
High	12 (35)	8 (21)	10 (26)	0.788^e^
MSKCC group				
1	4 (12)	4 (11)	10 (26)	
2	11 (32)	18 (47)	20 (53)	
3	19 (56)	16 (42)	8 (21)	0.083^f^
Nottingham/Barcelona score				
0	3 (9)	4 (11)	4 (11)	
1	8 (24)	8 (21)	21 (55)	
2	17 (50)	18 (47)	9 (24)	0.006^g^
3	6 (18)	8 (21)	4 (11)	
MTX cumulative dosage (g/m^2^)	–	23 [20–37]^h^	15 [9.5–21]	<0.001
MTX cycles	–	6 [5–8]^h^	5 [4–6]	<0.001
Mean MTX dosage in each cycle (g/m^2^)	–	3.8 [3.5–5]^h^	3.2 [3–3.5]	<0.001
WBRT doses (grays)				
Tumor bed	–	45 [40–50]^i^	40.25 [36–46]^j^	0.060
Whole brain	–	30 [28–40]^i^	30 [24–36]^j^	0.181
Response (before frontline intensification)				
CR/CRu	–	–	10/7 (26/18)	
PR	–	–	21 (55)	
Best response after completing induction therapy (including frontline intensification)				
CR/CRu	–	31/5 (82/13)	25/7 (66/18)	0.262
PR	–	2 (5)	6 (16)	

*Note*: –, not applicable; CR, complete remission; CRu, unconfirmed complete remission; DLBCL, diffuse large B‐cell lymphoma; ECOG, Eastern Cooperative Oncology Group performance score; IELSG, International Extranodal Lymphoma Study Group; LDH, lactate dehydrogenase; MSKCC, Memorial Sloan Kettering Cancer Center; MTX, methotrexate; NB, Nottingham/Barcelona; PCNSL, primary central nervous system lymphoma; PR, partial response; WBRT, whole‐brain radiotherapy.

^a^ Values are reported as median [interquartile range] or *n* (%).

^b^ Induction‐completed PCNSL patients with and without frontline intensification were compared; *p*‐values were determined using Mann–Whitney *U* tests for quantitative data and Fisher exact tests for categorical data.

^c^ Determined by patients with ECOG ≦ 1.

^d^ One patient received deceased‐donor kidney transplantation due to end‐stage renal disease related to chronic glomerulonephritis approximately 15 months before PCNSL diagnosis.

^e^ Determined by high‐risk IELSG.

^f^ Determined by MSKCC group 3.

^g^ Determined by Nottingham/Barcelona score with two or three points.

^h^ Determined by 23 patients receiving merely MTX‐based chemotherapy without frontline intensification.

^i^ Determined by 15 patients receiving mainly WBRT without frontline intensification.

^j^ Determined by 30 patients receiving WBRT or “WBRT and HDC‐ASCT” as frontline intensification.

Regarding ECOG performance and IELSG risk, there were no significant differences between induction‐completed patients who received frontline intensification and those who did not (Table 1). Regarding the cumulative dosage of MTX, patients who did not receive frontline intensification had significantly higher dosages than those who received frontline intensification (23 g/m^2^ vs. 17.5 g/m^2^, *p* < 0.001). Similarly, patients who did not receive frontline intensification had a trend of higher cumulative radiation dosage in the tumor bed than those who received frontline intensification (45 grays vs. 40 grays, *p* = 0.060).

### 3.3 Improved survival in PCNSL patients who received frontline intensification

**FIGURE 2:**
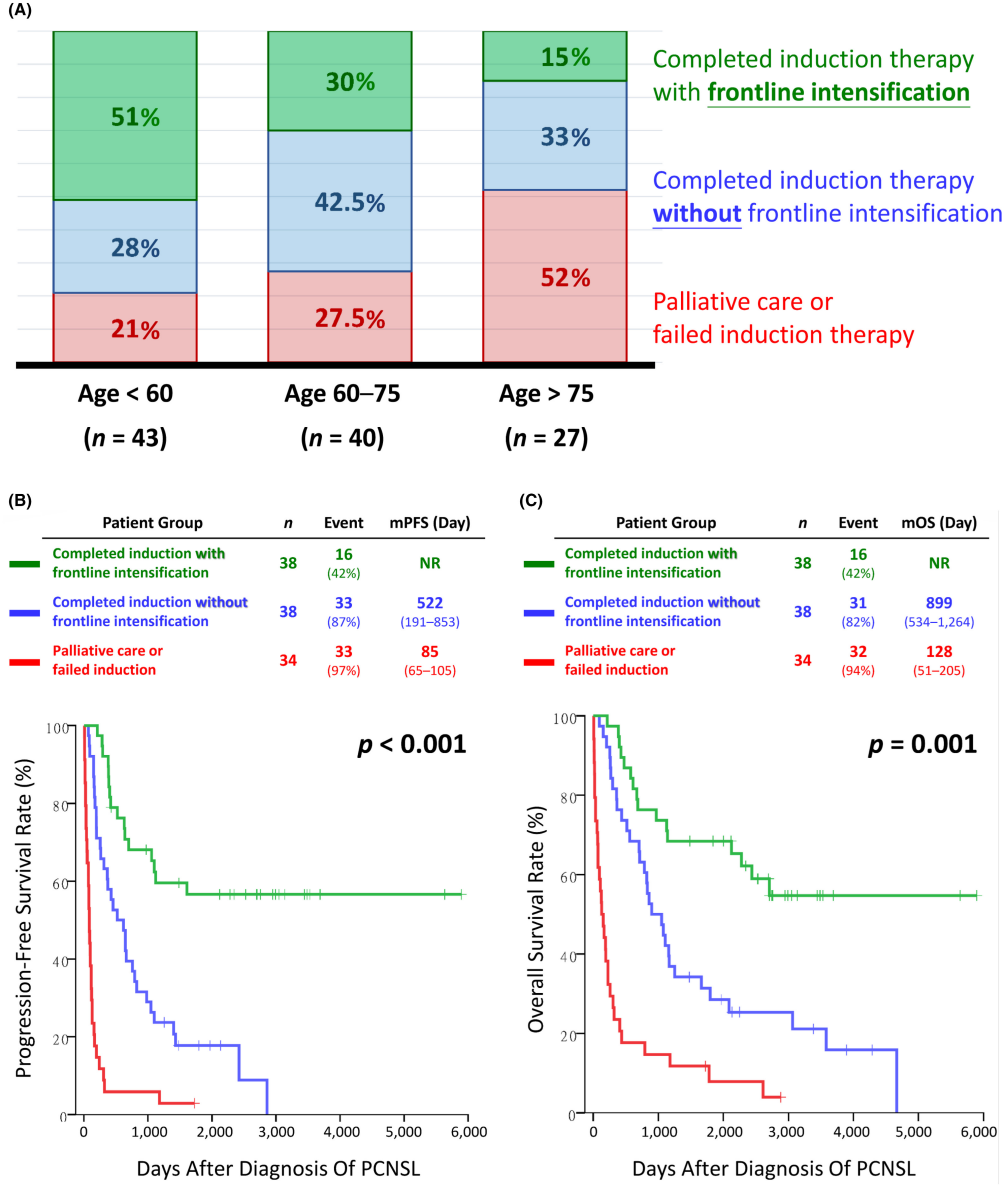
(A) Patients' percentage of completing induction with frontline intensification, completing induction without frontline intensification, and palliative care or induction failure in different age groups. (B) Progression‐free survival and (C) overall survival of 110 PCNSL patients subcategorized into three treatment groups; *p*‐values being determined by 76 PCNSL patients who completed induction therapy with and without frontline intensification. mOS, median overall survival; mPFS, median progression‐free survival; *n*, patient number; PCNSL, primary central nervous system lymphoma.

Table 1 shows that induction‐completed patients with frontline intensification had the lowest median age, and patients who chose palliative care or who failed their induction therapy were the oldest. As illustrated in Figure 2A, the younger group (age < 60) had a much higher percentage of patients who completed induction therapy with frontline intensification than the elderly group (age > 75); however, 21% of the patients in the younger group still failed their induction therapy or chose palliative care.

More importantly, induction‐completed PCNSL patients who received frontline intensification had a much longer median PFS (Figure 2B, not reached vs. 522 days, *p* < 0.001) and OS (Figure 2C, not reached vs. 899 days, *p* = 0.001) than those who did not receive frontline intensification. Among the 68 PCNSL patients who achieved CR/CRu after frontline therapy, patients who had frontline intensification consistently had a significantly better median PFS and OS than those without frontline intensification (Figure S2).

### 3.4 Outcome of PCNSL patients who received different modalities of frontline intensification

**FIGURE 3:**
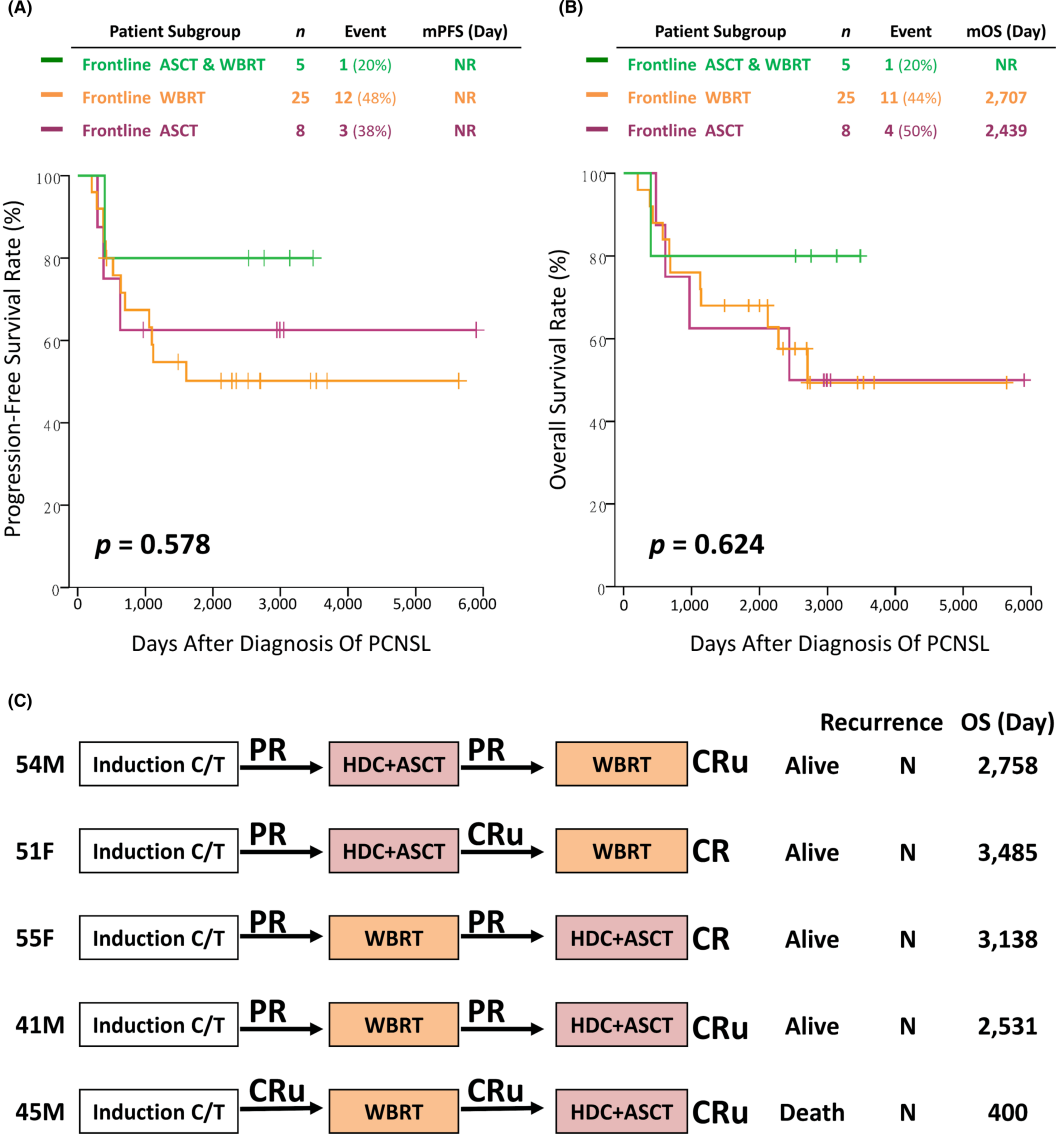
(A) Progression‐free survival and (B) overall survival of 38 induction‐completed PCNSL patients subcategorized according to three different modalities of frontline intensification. (C) Treatment response of five PCNSL patients who received tandem frontline intensification. C/T, chemotherapy; CR, complete remission; CRu, unconfirmed complete remission; HDC + ASCT, high‐dose chemotherapy with autologous stem‐cell transplant; *n*, number of patients; *N*, no; OS, overall survival; PR, partial remission; WBRT, whole‐brain radiotherapy.

When subcategorizing induction‐completed patients who received frontline intensification into three subgroups, patients receiving HDC‐ASCT or WBRT had similar PFS and OS rates (Figure 3A,B).

Of note, five patients received both WBRT and HDC‐ASCT as frontline intensification (Figure 3C), and four patients achieved a long‐term durable response. Before initial frontline intensification, four patients' treatment responses were PR, and only one patient's treatment response was CRu. After tandem WBRT and HDC‐ASCT frontline intensification, all five patients' treatment responses achieved CR/CRu.

### 3.5 Frontline intensification as an independent and favorable prognostic factor for induction‐completed PCNSL patients

IELSG risk, MSKCC group, and NB score are three prestigious prognostic scoring systems for PCNSL risk stratification at initial diagnosis,^11^, ^12^, ^13^ and we wonder if frontline intensification could improve poor prognostic factors. Regarding the 76 PCNSL patients who completed induction therapy, four models utilizing multivariate Cox regression analysis were adopted, as shown in Table S2.

In model I, sex, age ≥ 70, ECOG ≥2, elevated serum LDH, deep brain involvement, multiple‐site involvement, and frontline intensification were included, and factors with *p* < 0.2 in the univariate analysis were entered into the multivariate regression model, which indicates that frontline intensification remained an independent factor to predict both better PFS and better OS. We further examined the clinical impact of frontline intensification by adjusting it with IELSG intermediate‐ and high‐risk (model II), MSKCC groups 2 and 3 (model III), or NB scoring system of 2 and 3 points (model IV), and frontline intensification still significantly benefited both PFS and OS.

### 3.6 Survival benefit in different risk‐stratified PCNSLs provided by frontline intensification

**FIGURE 4:**
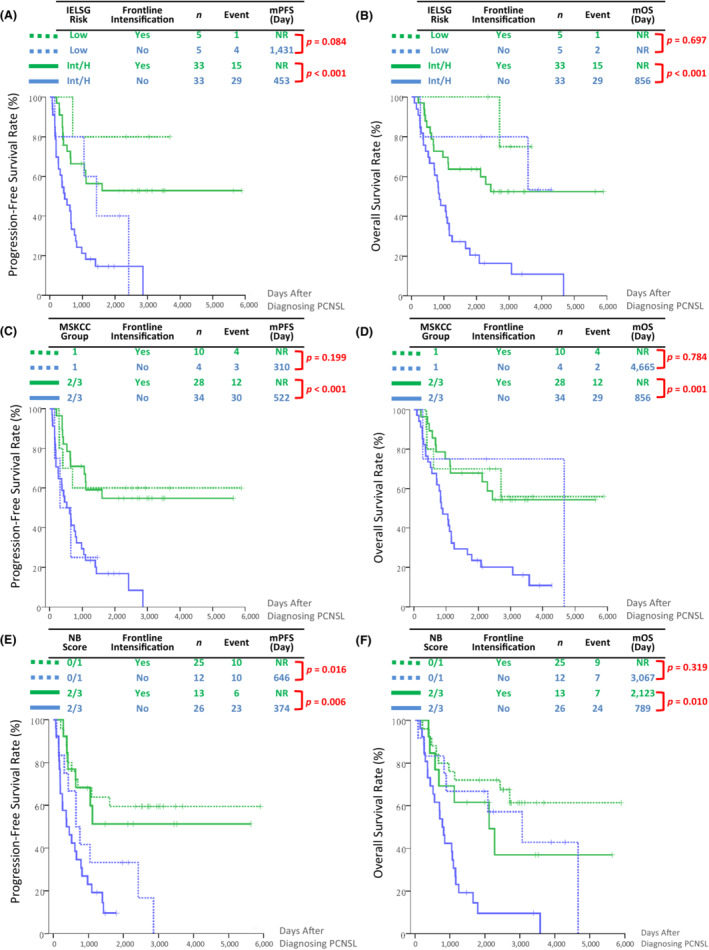
Progression‐free and overall survival in PCNSL patients stratified by with or without frontline intensification and different risk‐stratified systems, including IELSG risk (A, B), MSKCC group (C, D), and NB score (E, F). IELSG, International Extranodal Lymphoma Study Group; mOS, median overall survival; mPFS, median progression‐free survival; MSKCC, Memorial Sloan Kettering Cancer Center; NB, Nottingham/Barcelona.

For PCNSL, there is currently no existing treatment algorithm based on any risk stratification. Here we elucidate the survival benefit of frontline intensification in differently risk‐stratified PCNSL patients who completed induction therapy. In higher‐risk patients, both PFS and OS were significantly improved by frontline intensification in patients with intermediate or high IELSG risk (Figure 4A,B), MSKCC group 2 and 3 patients (Figure 4C,D), and patients with an NB score of ≥2 points (Figure 4E,F). In contrast, frontline intensification shows a trend of better PFS but is unable to improve OS In lower‐risk patients (low IELSG risk, MSKCC group 1, and NB score ≤ 1 point) (Figure 4). Interestingly, when PCNSL patients with higher risk (intermediate or high IELSG risk, MSKCC group 2 or 3, and NB score of ≥2 points) received frontline intensification, they achieved a similar PFS and OS to patients with lower risk (Figure S3).

### 3.7 Recurrence and salvage therapy after frontline intensification

**FIGURE 5:**
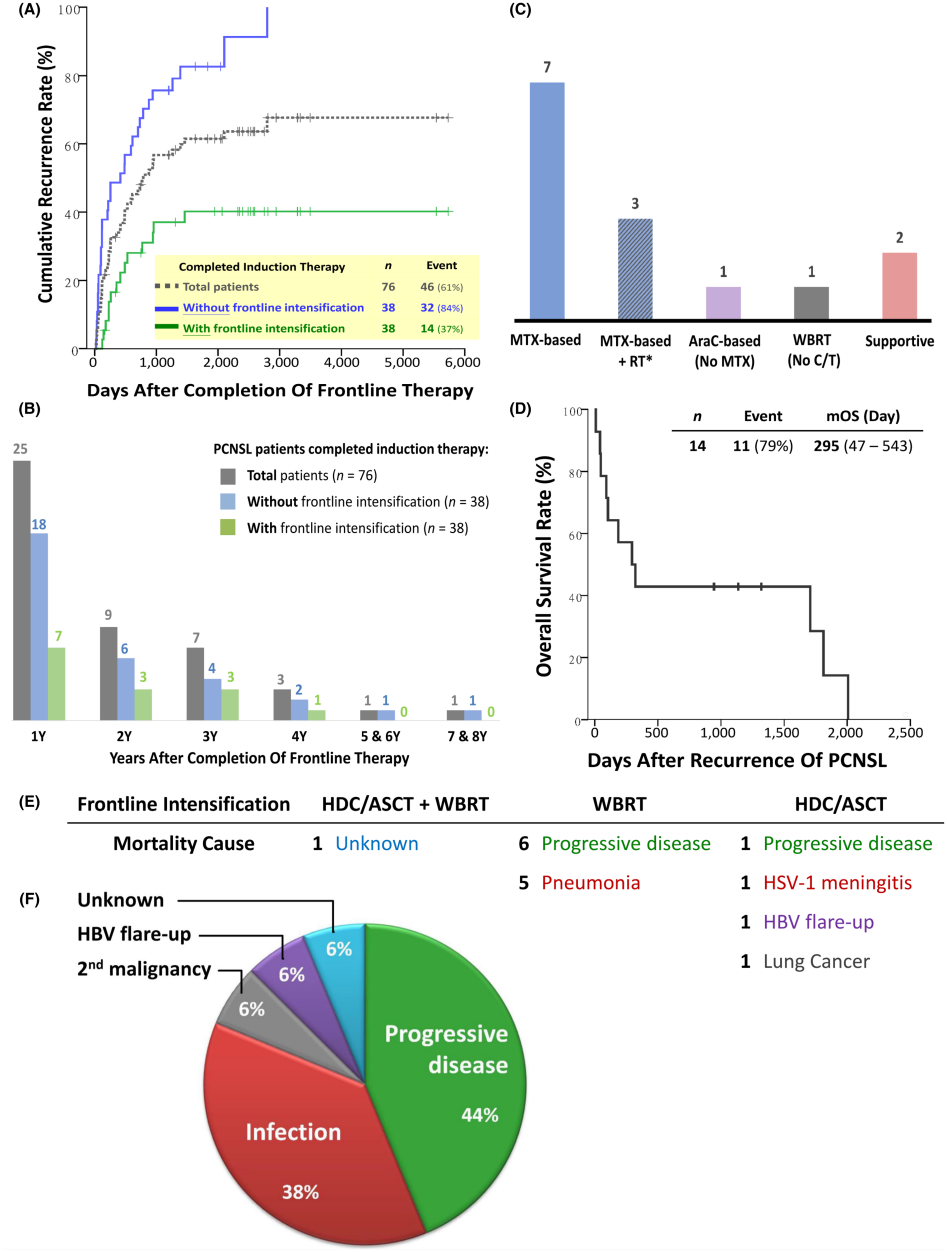
(A) Cumulative recurrence rate and (B) recurrent number of patients within each year after completion of frontline therapy among 76 induction‐completed PCNSL patients with or without frontline intensification. (C) Salvage therapy and (D) median overall survival of 14 PCNSL patients relapsed after frontline intensification; *One patient received whole‐brain RT, and two patients received local RT. (E and F) Causes of mortality for 16 induction‐completed PCNSL patients who received frontline intensification. AraC, cytarabine; C/T, chemotherapy; HBV, hepatitis B; HSV‐1, herpes simplex virus 1; mOS, median overall survival; MTX, methotrexate; *N*, patient number; PCNSL, primary central nervous system lymphoma; RT, radiotherapy; WBRT, whole‐brain radiotherapy; Y, years.

In the 76 induction‐completed PCNSL patients, most recurrence (89%) took place within the first 3 years after completion of frontline therapy, and more than half (54%) of the recurrences occurred within 1 year after completing frontline therapy (Figure 5A,B). Among the 38 induction‐completed patients who received frontline intensification, 14 had recurrent PCNSL. In contrast, PCNSL almost inevitably recurred in the other 38 induction‐completed patients, who did not receive frontline intensification, and some recurred even 5 years after completion of induction therapy.

In the 14 patients who recurred after frontline intensification, rechallenging with high‐dose methotrexate combined with rituximab and other chemotherapy remained the major salvage regimen (Figure 5C). However, the overall prognosis was dismal, and the median overall survival was as short as 295 days after recurrence (Figure 5D).

### 3.8 Causes of mortality among PCNSL patients who received frontline intensification

Among the 38 induction‐completed patients who received frontline intensification, 16 died (Figure 5E,F). Two cases could be attributed to treatment‐related mortality: one case had aspirational pneumonia with respiratory failure shortly after completing WBRT; another patient started to suffer from diplopia and unsteady gait 2 months after tandem frontline intensification (WBRT followed by HDC‐ASCT) and died 5 months after tandem frontline intensification; restaging workup (including brain MRI and CSF analysis from lumbar puncture) revealed no evidence of recurrent PCNSL, and unknown encephalopathy related to PCNSL treatment is suspected. Otherwise, progressive disease and infection (especially pneumonia) were two major etiologies of mortality. Of note, one patient died of a second malignancy, and another patient had acute liver decompensation due to hepatitis B flare‐up nearly 5 years after salvage methotrexate and WBRT.

## 4 DISCUSSION

Here we elucidate the clinical benefits of frontline intensification in real‐world PCNSL patients and highlight several important findings. First, our study shows a prominent escalation in the CR/CRu rate after frontline intensification. Such an elevated CR/CRu rate reflects therapeutic outcomes of real‐world PCNSL patients and is consistent with recent trials indicated by IELSG‐32^6^, ^7^ and PRECIS,^8^ as shown in Table S3. In fact, frontline intensification, especially HDC‐ASCT, was not a popular or widely accepted concept for treating real‐world PCNSL in Taiwan before 2010, as most induction‐completed cases would not have received frontline intensification if they achieved CR/CRu during initial induction therapy, and some physicians tended to give patients more cycles of MTX‐based regimens or higher dosage of MTX instead of arranging frontline intensification. In contrast, the majority of patients who received frontline intensification were those who achieved unsatisfactorily PR after induction therapy and therefore received subsequent intensification therapy attempting to improve treatment response.

Second, while WBRT is traditionally considered the most powerful consolidative therapy, its neurological sequela drive clinicians to search for alternative strategies, especially considering the 100% delayed neurotoxicity incidence rate in patients older than age 60 who have undergone standard dose radiotherapy.^17^ Increasing evidence shows convincible efficacy with HDC‐ASCT: the IELSG‐32 trial revealed a comparable response between WBRT and HDC‐ASCT,^6^ and the PRECIS trial even showed a favorable trend of PFS in the HDC‐ASCT arm.^8^ Our real‐world cohort demonstrated a similar treatment response between WBRT and HDC‐ASCT (Figure 3A,B), and each arm of frontline intensification achieved two‐year PFS and OS rates of at least 60% and 70%, respectively.

Third, conditioning regimens for ASCT could be separated into thiotepa‐based and non‐thiotepa regimens. There have been no head‐to‐head randomized trials to compare different regimens, but the thiotepa‐based regimen has demonstrated a superior response rate and duration in many single‐arm trials.^2^, ^3^, ^4^, ^18^, ^19^ Thirteen PCNSL patients in our cohort received frontline HDC‐ASCT, including eight for BEAM (carmustine [BCNU], etoposide, cytarabine, melphalan), three for thiotepa plus BCNU, and two for BEAC (BCNU, etoposide, cytarabine, cyclophosphamide). Given that the National Health Insurance (NHI) program in our country covers the cost of BCNU, but not thiotepa, the majority adopted the BEAM regimen, and half of them saw long‐term survival. In a recent retrospective study that used data from the Center for International Blood and Marrow Transplant Research registry,^20^ the thiotepa‐based conditioning regimen was associated with higher PFS and OS compared with BEAM, and these inspiring results will help us to push for including thiotepa for PCNSL patients in Taiwan's NHI program.

Fourth, PCNSL in the elderly remains a challenge as these patients may be precluded from sufficient treatment intensity and receiving frontline intensification. A recent large retrospective study from the French LOC network clearly indicated that consolidation treatments were administered in 77% of patients <60 years old but only in 11% of patients >60 years old.^21^ Consistently, our cohort demonstrated that only 30% and 15% of patients between ages 60 and 75, and more than age 75, respectively, received frontline intensification.

Fifth, patients in our cohort who did not receive frontline intensification inevitably recurred, which reflects not only insufficient treatment intensity but also the unsatisfactory quality of response, given that more than 90% of these patients achieved CR/CRu after induction therapy. Positron emission tomography with fluorine‐18 fluorodeoxyglucose has an intrinsic defect in delineating lesions within the brain parenchyma, and gadolinium‐enhanced magnetic resonance imaging shows relevant limitations in detecting subtle residual tumors; therefore, there is a limitation with imaging tools for evaluating treatment response. In contrast, detecting circulating tumor DNA in CSF or serum by PhasED sequencing could be a novel strategy for outcome prediction and monitoring of measurable residual disease in PCNSL patients.^22^

Our study has several limitations. It's a retrospective study trying to elucidate the survival benefits of frontline intensification in real‐world PCNSL patients, and we have tried our best to adjust for measurable confounding factors for reducing biases; in fact, there has not been any randomized control trial capable of determining the survival benefit provided by frontline intensification. Our study did not cover the impact of frontline intensification on neurotoxicity and patients' cognitive function, because many earlier cases did not complete comprehensive neurological assessment; however, those having received frontline HDC‐ASCT maintained mostly acceptable cognitive function and quality of life in our cohort (data not shown), and we believe that tumor recurrence outweighs the neurotoxicity brought on by frontline intensification in causing deterioration of CNS function.

In conclusion, PCNSL is a rare and dismal phenotype of aggressive NHL. Our study bridges the gap in the current evidence of consolidative WBRT or HDC‐ASCT for treating PCNSL. We indicate that frontline intensification can improve both PFS and OS in higher‐risk PCNSL patients, including those with intermediate/high IELSG risk, MSKCC group 2/3, and Nottingham/Barcelona score ≥ 2 points. Consequently, our study strengthens the evidence for adopting frontline intensification in PNCSL patients if they are physically fit and have completed induction therapy achieving at least PR, especially those with higher‐risk PCNSL.

## AUTHOR CONTRIBUTIONS

**Hao‐Yuan Wang:** Conceptualization (lead); data curation (lead); formal analysis (lead); funding acquisition (equal); investigation (equal); methodology (lead); project administration (equal); resources (equal); visualization (lead); writing – original draft (lead); writing – review and editing (lead). **Ching‐Fen Yang:** Data curation (supporting); formal analysis (supporting); investigation (equal); methodology (supporting). **Chia Hsin Lin:** Data curation (supporting); methodology (supporting); validation (equal). **Liang‐Tsai Hsiao:** Resources (equal). **Po‐Shen Ko:** Resources (equal). **Yao‐Chung Liu:** Resources (equal). **Tzeon‐Jye Chiou:** Resources (equal). **Po‐Min Chen:** Supervision (supporting). **Jyh‐Pyng Gau:** Resources (equal); supervision (lead); validation (supporting). **Jin‐Hwang Liu:** Resources (equal); supervision (supporting); validation (supporting). **Chia‐Jen Liu:** Funding acquisition (equal); project administration (equal); resources (equal); validation (equal); writing – review and editing (equal).

## CONFLICT OF INTEREST

The authors declare that they have no conflict of interest.

## Supporting information


Table S1.
Table S2.
Table S3.
Figure S1.
Figure S2.
Figure S3.

## Data Availability

The datasets used and analyzed in this study are available from the corresponding author on reasonable request.
