# Supplementary material for: Long‐term outcomes of frontline intensification in primary CNS lymphoma: A real‐world single‐center experience
Source: Cancer Med. 2023 Jan 17;12(7):8089–101. doi: 10.1002/cam4.5607 (PMC10134300; doi:10.1002/cam4.5607)
Supplement: Supplementary file 1 — Table S1. Table S2. Table S3. Figure S1. Figure S2. Figure S3. [file CAM4-12-8089-s001.pdf]

Supplementary Data

Long-term outcomes of frontline intensification in primary CNS lymphoma: A real-world single-center experience

Running title: Frontline intensification for PCNSL

Hao-Yuan Wang,[1,2] Ching-Fen Yang,[2,3] Chia-Hsin Lin,[4] Liang-Tsai Hsiao,[1,2] Po-Shen Ko,[1,2] Yao-Chung Liu,[1,2] Tzeon-Jye Chiou,[1,2] Po-Min Chen,[1,2] Jyh-Pyng Gau,[1,2] Jin-Hwang Liu,[1,2,5,6] Chia-Jen Liu[1,2,7]

[1]Division of Hematology and Oncology, Department of Medicine, Taipei Veterans General Hospital, Taipei, Taiwan

[2]Faculty of Medicine, School of Medicine, National Yang Ming Chiao Tung University, Taipei, Taiwan

[3]Department of Pathology and Laboratory Medicine, Taipei Veterans General Hospital, Taipei, Taiwan

[4]Department of Radiation Oncology, Linkou Chang Gung Memorial Hospital Medical Center, Taoyuan City, Taiwan

[5]Institute of Biopharmaceutical Sciences, National Yang Ming Chiao Tung University, Taipei, Taiwan

[6]Chong Hin Loon Memorial Cancer and Biotherapy Research Center, National Yang Ming Chiao Tung University, Taipei, Taiwan

[7]Institute of Public Health, National Yang Ming Chiao Tung University, Taipei, Taiwan

Table of contents		Page
**Table S1**	Clinical characteristics of 110 intention-to-treat PCNSL patients	**2**
**Table S2**	Univariate and multivariate analyses of factors predicting survival in 76 PCNSL patients who completed induction therapy	**3**
**Table S3**	Summary of IELSG-32, PRECIS, and our cohort utilizing WBRT and/or HDC-ASCT as consolidation	**4**
**Figure S1**	(A) Progression-free survival and (B) overall survival among 110 intention-to-treat PCNSL patients	**5**
**Figure S2**	(A) Progression-free survival and (B) overall survival of 68 PCNSL patients who completed frontline therapy achieved CR/CRu.	**6**
**Figure S3**	Compare the progression-free and overall survival between higher-risk PCNSL patients who received frontline intensification and lower-risk PCNSL patients stratified by three different prognostic systems, including IELSG risk (A and B), MSKCC group (C and D), and NB score (E and F).	**7-8**

Table S1. Clinical characteristics of 110 intention-to-treat PCNSL patients

Patient characteristics	No. of patients (%). Median of value [interquartile-range]
Age, years	65 [54–74]
< 70	70 (64)
≥ 70	40 (36)
Sex, male	60 (55)
ECOG performance	
0	17 (15)
1	34 (31)
2	26 (24)
3	22 (20)
4	11 (10)
Histology: DLBCL	110 (100)
PTLD	1 (1)
Elevated serum LDH	52 (47)
Multiple brain lesions [a]	55 (50)
Deep-brain involvement [b]	74 (67)
Ocular involvement	14 (13)
Leptomeningeal involvement [c]	4 (4)
IELSG risk	
Low	10 (9)
Intermediate	70 (64)
High	30 (27)
MSKCC group	
1	18 (16)
2	49 (45)
3	43 (39)
Nottingham/Barcelona score	
0	11 (10)
1	37 (34)
2	44 (40)
3	18 (16)

[a] Defined as two or more lesions
[b] Defined as periventricular regions, basal ganglia, brainstem, and/or cerebellum
[c] Proved by cytology from CSF fluid

DLBCL, diffuse large B-cell lymphoma; ECOG, Eastern Cooperative Oncology Group performance score; LDH, lactate dehydrogenase; IELSG, International Extranodal Lymphoma Study Group; MSKCC, Memorial Sloan Kettering Cancer Center; PCNSL, primary central nervous system lymphoma; PTLD, post-transplant lymphoproliferative disease

Table S2. Univariate and multivariate analyses of factors predicting survival in 76 PCNSL patients who completed induction therapy

Clinical factors	Progression-free survival				Overall survival			
**Model I:**	**Univariate**		**Multivariate [a,b,c]**		**Univariate**		**Multivariate [a,b,c]**	
	HR (95% CI)	*p*	HR (95% CI)	*p*	HR (95% CI)	*p*	HR (95% CI)	*p*
Age ≥ 70	1.546 (0.853–2.800)	0.151			1.827 (1.006–3.316)	0.048		
Male sex	1.359 (0.764–2.420)	0.297			1.579 (0.868–2.871)	0.134		
ECOG ≥ 2	1.797 (1.019–3.170)	0.043	1.670 (0.939–2.969)	0.081	2.177 (1.206–3.928)	0.010	2.233 (1.224–4.073)	0.009
Elevated serum LDH	1.012 (0.572–1.789)	0.968	–	–	1.093 (0.611–1.955)	0.765	–	–
Deep brain involvement	1.084 (0.605–1.942)	0.787	–	–	1.211 (0.661–2.218)	0.535	–	–
Multiple-site involvement	1.136 (0.647–1.996)	0.656	–	–	1.153 (0.648–2.053)	0.627	–	–
**Frontline intensification**	**0.283 (0.154–0.522)**	**<0.001**	**0.291 (0.157–0.538)**	**<0.001**	**0.358 (0.194–0.659)**	**0.001**	**0.350 (0.189–0.650)**	**0.001**
**Model II:**	**Univariate**		**Multivariate [b,d]**		**Univariate**		**Multivariate [b,d]**	
	HR (95% CI)	*p*	HR (95% CI)	*p*	HR (95% CI)	*p*	HR (95% CI)	*p*
IELSG risk (int. or high)	1.903 (0.753–4.812)	0.174	2.149 (0.846–5.459)	0.108	3.595 (1.100–11.748)	0.034	4.103 (1.241–13.573)	0.021
**Frontline intensification**	**0.283 (0.154–0.522)**	**<0.001**	**0.272 (0.147–0.503)**	**<0.001**	**0.358 (0.194–0.659)**	**0.001**	**0.335 (0.181–0.619)**	**<0.001**
**Model III:**	**Univariate**		**Multivariate [b,d]**		**Univariate**		**Multivariate [b,d]**	
	HR (95% CI)	*p*	HR (95% CI)	*P*	HR (95% CI)	*p*	HR (95% CI)	*p*
MSKCC group (2 or 3)	1.479 (0.664–3.297)	0.338	–	–	2.041 (0.853–4.881)	0.109		
**Frontline intensification**	**0.283 (0.154–0.522)**	**<0.001**	**0.283 (0.154–0.522)**	**<0.001**	**0.358 (0.194–0.659)**	**0.001**	**0.358 (0.194–0.659)**	**0.001**
**Model IV:**	**Univariate**		**Multivariate [b,d]**		**Univariate**		**Multivariate [b,d]**	
	HR (95% CI)	*p*	HR (95% CI)	*P*	HR (95% CI)	*p*	HR (95% CI)	*p*
NB score (2 or 3 points)	1.953 (1.098–3.473)	0.023			2.841 (1.537–5.251)	0.001	2.397 (1.275–4.507)	0.007
**Frontline intensification**	**0.283 (0.154–0.522)**	**<0.001**	**0.283 (0.154–0.522)**	**<0.001**	**0.358 (0.194–0.659)**	**0.001**	**0.427 (0.228–0.798)**	**0.008**

[a] The backward method is determined for the multivariate Cox regression model due to the small number of cases
[b] Multivariate Cox regression model included all available variables with $p \leqq 0.200$
[c] Age and sex are forced into the multivariate analysis because they may confound between-subject comparisons
[d] Enter method is determined for the multivariate Cox regression model due to only two competing factors

–, not entered into the multivariate regression model; CI, confidence interval; ECOG, Eastern Cooperative Oncology Group performance score; HR, hazard ratio; IELSG, International Extranodal Lymphoma Study Group score; int., intermediate; LDH, lactate dehydrogenase; MSKCC, Memorial Sloan Kettering Cancer Center; NB, Nottingham/Barcelona

Table S3: Summary of IELSG-32, PRECIS, and our cohort utilizing WBRT and/or HDC-ASCT as consolidation

Trial	Subgroup	No. of patients	Age (Y)	Male	ECOG > 1	Multiple	IELSG (L/I/H)	CR/CRu		2Y PFS	2Y OS
								Before consolidation	After consolidation		
**IELSG-32**	**Group D [a]**	59	58	69%	19%	53%	15/73/12%	54%	95%	80%	85%
	**Group E [a]**	59	58	51%	34%	58%	25/61/14%	53%	93%	69%	71%
**PRECIS**	**WBRT [b]**	38	53	71%	34%	55%	–	53%	71%	63%	86%
	**ASCT [b]**	38	55	63%	26%	63%	–	45%	82%	87%	86%
**TPE-VGH**	**ASCT**	8	56	63%	38%	38%	13/75/13%	38%	75%	63%	71%
	**WBRT**	25	65	44%	44%	60%	16/52/32%	52%	84%	67%	76%
	**WBRT+ASCT**	5	51	60%	20%	40%	0/80/20%	20%	100%	80%	80%
	**Total**	38	57	50%	39%	53%	13/61/26%	45%	84%	68%	76%

[a] Denoting the intent-to-treat population of the IELSG-32 trial, patients in Group D were those being randomized to consolidative WBRT; patients in Group E were those being randomized to consolidative HDC-ASCT.
[b] Denotes the primary-end-point population of the PRECIS trial, which meant the first 38 patients who completed the consolidation in each arm.

–, not analyzed; ASCT, high-dose chemotherapy with autologous stem-cell transplant; CR, complete remission; CRu, unconfirmed complete remission; ECOG, Eastern Cooperative Oncology Group performance score; IELSG, International Extranodal Lymphoma Study Group; L/I/H, low/intermediate/high risk; No., number; OS, overall survival; PFS, progression-free survival; TPE-VGH, Taipei Veterans General Hospital; WBRT, whole-brain radiotherapy; Y, year

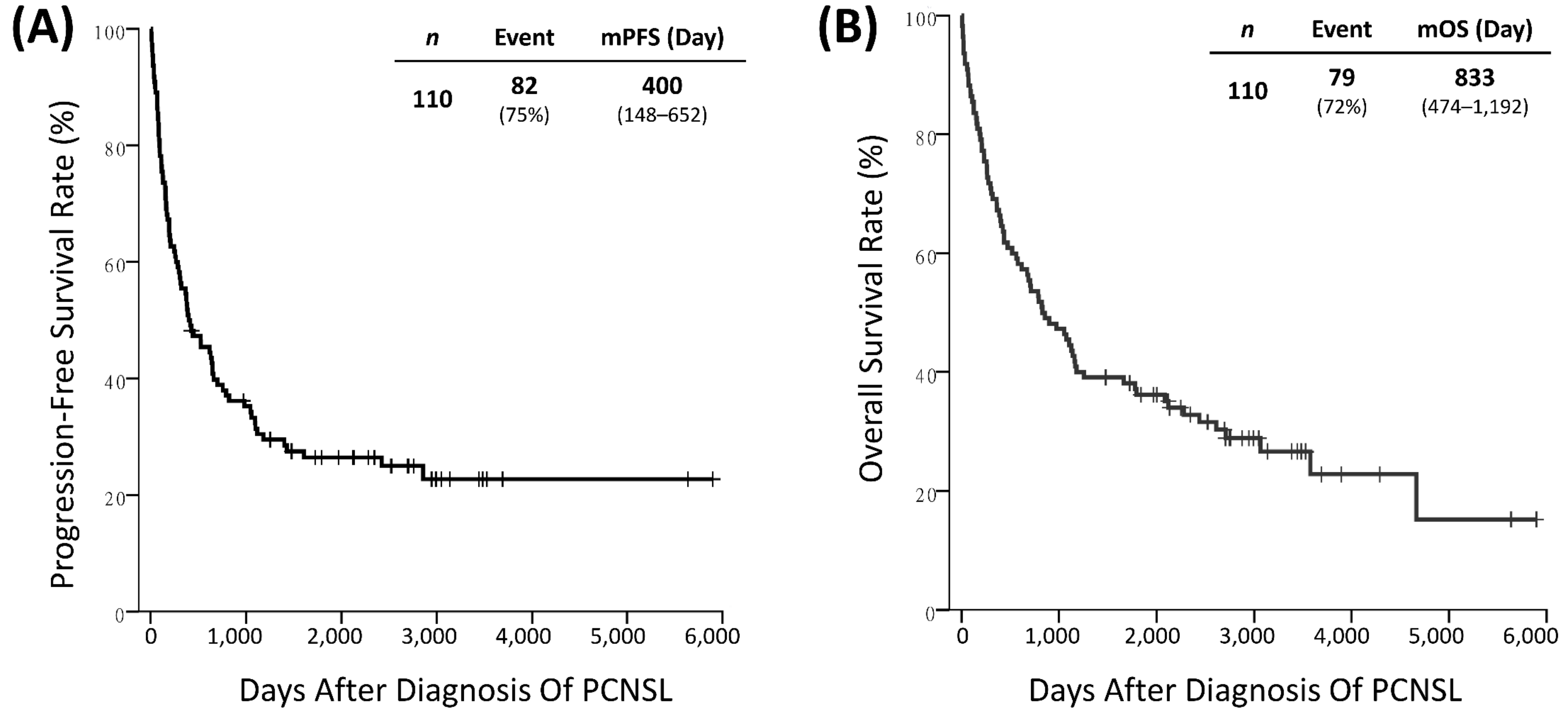

Figure S1. (A) Progression-free survival and (B) overall survival among 110 intention-to-treat PCNSL patients

mOS, median overall survival; mPFS, median progression-free survival; N, number of patients; PCNSL, primary central nervous system lymphoma.

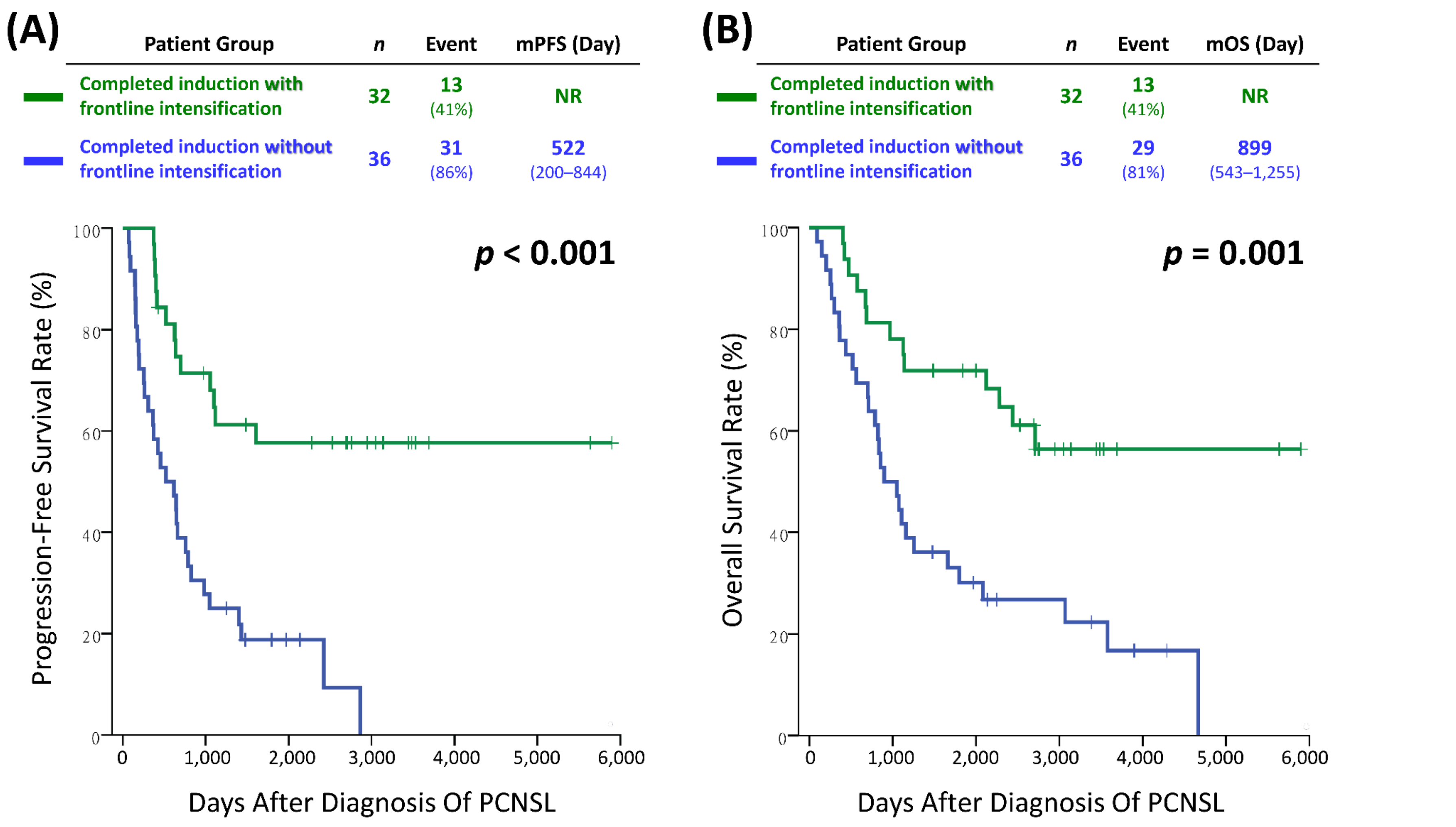

Figure S2. (A) Progression-free survival and (B) overall survival of 68 PCNSL patients who completed frontline therapy achieved CR/CRu.

CR, complete remission; CRu, unconfirmed complete remission; mOS, median overall survival; mPFS, median progression-free survival; *n*, patient number; PCNSL, primary central nervous system lymphoma

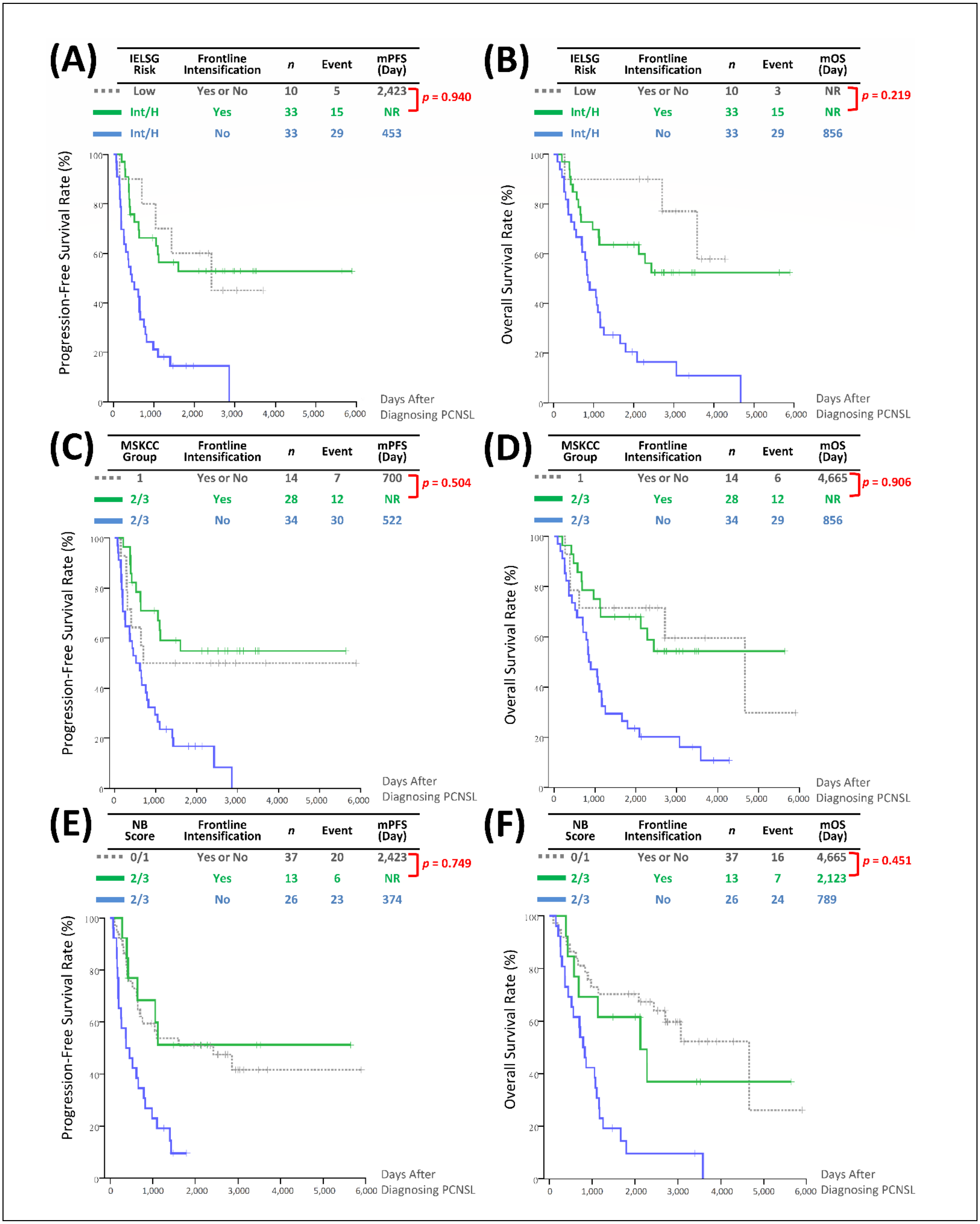
(A)
IELSG Risk | Frontline Intensification | n | Event | mPFS (Day)
Low | Yes or No | 10 | 5 | 2,423
Int/H | Yes | 33 | 15 | NR
Int/H | No | 33 | 29 | 453
p = 0.940
Progression-Free Survival Rate (%)
Days After Diagnosing PCNSL
(B)
IELSG Risk | Frontline Intensification | n | Event | mOS (Day)
Low | Yes or No | 10 | 3 | NR
Int/H | Yes | 33 | 15 | NR
Int/H | No | 33 | 29 | 856
p = 0.219
Overall Survival Rate (%)
Days After Diagnosing PCNSL
(C)
MSKCC Group | Frontline Intensification | n | Event | mPFS (Day)
1 | Yes or No | 14 | 7 | 700
2/3 | Yes | 28 | 12 | NR
2/3 | No | 34 | 30 | 522
p = 0.504
Progression-Free Survival Rate (%)
Days After Diagnosing PCNSL
(D)
MSKCC Group | Frontline Intensification | n | Event | mOS (Day)
1 | Yes or No | 14 | 6 | 4,665
2/3 | Yes | 28 | 12 | NR
2/3 | No | 34 | 29 | 856
p = 0.906
Overall Survival Rate (%)
Days After Diagnosing PCNSL
(E)
NB Score | Frontline Intensification | n | Event | mPFS (Day)
0/1 | Yes or No | 37 | 20 | 2,423
2/3 | Yes | 13 | 6 | NR
2/3 | No | 26 | 23 | 374
p = 0.749
Progression-Free Survival Rate (%)
Days After Diagnosing PCNSL
(F)
NB Score | Frontline Intensification | n | Event | mOS (Day)
0/1 | Yes or No | 37 | 16 | 4,665
2/3 | Yes | 13 | 7 | 2,123
2/3 | No | 26 | 24 | 789
p = 0.451
Overall Survival Rate (%)
Days After Diagnosing PCNSL

Figure S3. Compare the progression-free and overall survival between higher-risk PCNSL patients who received frontline intensification and lower-risk PCNSL patients stratified by three different prognostic systems, including IELSG risk (A and B), MSKCC group (C and D), and NB score (E and F).

IELSG, International Extranodal Lymphoma Study Group; mOS, median overall survival; mPFS, median progression-free survival; MSKCC, Memorial Sloan Kettering Cancer Center; NB, Nottingham/Barcelona; PCNSL, primary central nervous system lymphoma
